# *N*‐Heterocyclic Carbene Self‐assembled Monolayers on Copper and Gold: Dramatic Effect of Wingtip Groups on Binding, Orientation and Assembly

**DOI:** 10.1002/cphc.201701045

**Published:** 2017-11-02

**Authors:** Christian R. Larrea, Christopher J. Baddeley, Mina R. Narouz, Nicholas J. Mosey, J. Hugh Horton, Cathleen M. Crudden

**Affiliations:** ^1^ EaStCHEM School of Chemistry University of St Andrews St Andrews Fife United Kingdom; ^2^ Department of Chemistry Queen's University Chernoff Hall Kingston Ontario Canada; ^3^ Institute of Transformative Bio-Molecules (WPI-ITbM) Nagoya University Chikusa Nagoya Japan

**Keywords:** copper, *N*-heterocyclic carbenes, orientation, self-assembled monolayers, wingtip

## Abstract

Self‐assembled monolayers of *N*‐heterocyclic carbenes (NHCs) on copper are reported. The monolayer structure is highly dependent on the *N*,*N*‐substituents on the NHC. On both Cu(111) and Au(111), bulky isopropyl substituents force the NHC to bind perpendicular to the metal surface while methyl‐ or ethyl‐substituted NHCs lie *flat*. Temperature‐programmed desorption studies show that the NHC binds to Cu(111) with a desorption energy of *E*_des_=152±10 kJ mol^−1^. NHCs that bind *upright* desorb cleanly, while *flat‐lying* NHCs decompose leaving adsorbed organic residues. Scanning tunneling microscopy of methylated NHCs reveals arrays of covalently linked dimers which transform into adsorbed (NHC)_2_Cu species by extraction of a copper atom from the surface after annealing.

Control over the orientation of molecules within self‐assembled monolayers (SAMs) is critical. In thiolate SAMs on gold, solution deposition methods give *upright* binding modes through a two‐step process, with *flat‐lying* species present at low coverage transforming into dense *upright* SAMs at higher concentrations and extended times.^1^ The ability to prepare well‐defined SAMs with predictable properties (e.g. hydrophobicity/hydrophilicity) is a hallmark of thiol‐based SAMs and is critically dependent on molecular orientation^2^, ^3^

Recently, *N*‐heterocyclic carbenes (NHCs) have emerged as promising alternatives to thiols for the formation of robust, ordered SAMs on Au.^4^, ^5^, ^6^, ^7^, ^8^ Seminal work by Siemeling^4^ and Johnson^5^ showed that NHCs bind to planar Au surfaces. Crudden and Horton demonstrated that NHCs form clean, well‐ordered monolayers with exceptionally high stability, surviving treatment with boiling organic solvent, acid, base, oxidant,^6^ and high temperatures.^9^ These conditions would destroy typical thiol‐based SAMs.^10^, ^11^ Other studies of NHC films on Au have shown remarkable effects on work function,^12^ and highly ordered structures can be imaged by low temperature scanning tunneling microscopy (STM).^7^ In addition, the formation of strong C−Si bonds has recently been reported following NHC adsorption on Si(111).^13^

Despite the impact of these studies, the effect of NHC structure on SAM formation has barely been examined. Additionally, the ability of NHCs to form monolayers on other, more reactive metals has received no attention outside the realm of nanoparticles.^14^ Thiol‐based SAMs on more reactive metals such as Cu, Ag, Pt, Ni, etc. are prone to decomposition and generation of metal sulfides, creating a pressing need to find alternatives to S‐based ligands for these metals.^15^

Herein we present the preparation of highly ordered, thermally stable NHC films on Cu(111). Through STM, high resolution electron energy loss spectroscopy (HREELS), and temperature programmed desorption (TPD) studies, we demonstrate that small differences in the size of the NHC wingtip groups lead to two distinct binding modes. In addition, we find that NHCs bind to Cu(111) with a similarly high adsorption energy to that observed on Au(111).

Bench‐stable benzimidazolium bicarbonates bearing methyl, ethyl, or isopropyl wing tip groups (**1** to **3**) were vapor deposited onto clean Cu(111) and Au(111) surfaces (Figure 1 a). The resulting SAMs were examined by HREELS in the specular geometry where spectra are dominated by dipole scattering such that the surface dipole selection rule provides experimental information on adsorbate orientation.^16^, ^17^

**Figure 1:**
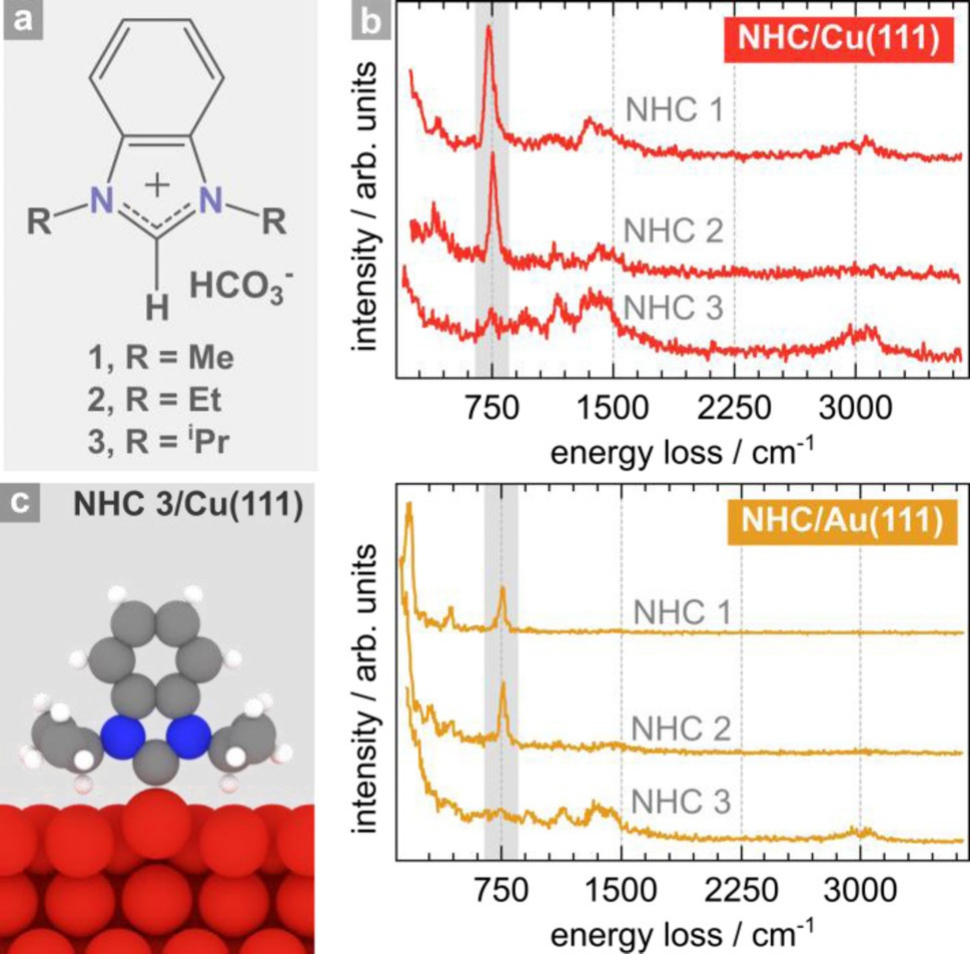
NHC‐based SAMs on Cu(111) and Au(111): a) Structure of NHC precursors. b) HREEL spectra of NHC monolayers derived from **1**–**3** examined on Cu(111) and Au(111) at 300 K. c) DFT‐optimized binding geometry of isopropyl‐substituted NHC **3** on Cu(111).

Spectra obtained following adsorption of NHCs **1** and **2** on both Cu(111) and Au(111) were dominated by a very strong peak at 730 cm^−1^, assigned to the out‐of‐plane aromatic C−H bending mode, whose dipole moment is normal to the molecular plane. Features at 2940 and 3075 cm^−1^ (assigned to the alkyl and aromatic ring C−H stretches, respectively) and at 1250–1600 cm^−1^ (C‐N and C=C stretches, and C−H bending modes) all appear very weak. The relative intensities of these energy losses provide strong evidence that the molecular planes of **1** and **2** are aligned approximately parallel to both the Au(111) and Cu(111) surface planes. (Figure 1 b).

Prolonged exposure of NHC **1** to Cu(111) led to significant enhancement of the weaker signals, implying an increasing amount of *upright* species at higher coverage (Figure S1). However, the 730 cm^−1^ peak was not suppressed; therefore, the spectrum reflects the coexistence of *upright* and *flat‐lying* species. Such behavior is not uncommon^18^ ‐a similar interpretation was proposed for the coverage‐dependence of benzoic acid adsorption on Cu(110).^19^

Spectra recorded for the bulkier NHC **3** on Cu(111) and Au(111) are significantly different from those obtained for NHCs **1** and **2**, with strong peaks observed with **1** and **2** appearing weak for **3**, and vice versa (Figure 1 b). NHC **3** must, therefore, adopt an upright geometry. This is consistent with the DFT optimized geometry (Figure 1 c), and analogous to results obtained from NHC **3** on Au(111).^6^, ^9^, ^12^

STM revealed the presence of highly‐ordered molecules of NHC **1** on Cu(111) at 300 K, along with trenches of a depth equivalent to a single Cu atom. Elliptical features of length 3.4±0.7 Å, were resolved in the trenches (Figure 2 a). Adjacent features were separated by 3.8±0.6 Å, consistent with individual *upright* species derived from NHC **1** stacked via intermolecular π‐π interactions.^20^

**Figure 2:**
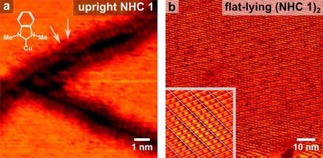
NHC‐based SAMs on Cu(111). STM image of: a) *upright* dimethyl NHC **1** on Cu(111) hosted in trenches as indicated by arrows; b) co‐existing *flat‐lying* SAM (4 −4|8 2) at 300 K; inset: Fourier‐filtered image of dimeric features comprising the SAM (1 −3|9 4), image size 9.9×9.9 nm^2^. Unit cell is marked by overlaid grid.

These *upright* species coexist with features assigned to *flat‐lying* molecules periodically arranged (Figure 2 b) into a superstructure consistent with a commensurate (4 −4|8 2) unit cell containing 40 Cu atoms and two dimeric features of length 0.91±0.09 nm which are tentatively ascribed to enetetramine species resulting from the dimerization of NHCs.^21^ Enetetramines were employed by Siemeling^4^ as potential precursors to NHC‐functionalized surfaces, but have never previously been observed intact on a surface.

The inset in Figure 2 b displays molecular features whose periodicity is consistent with a commensurate (1 −3|9 4) structure containing one enetetramine species per unit cell (31 Cu atoms). When imaging at 300 K, the islands fluctuated in shape due to the high mobility of individual species with images being acquired slightly below saturation coverage.

Annealing the sample to 365 K resulted in the disappearance of *upright* species and the formation of a new ordered molecular arrangement. Analysis of the Fourier transform of the image revealed unit cell vectors ***a*** **=**1.55 nm; ***b*** **=**2.43 nm, with an included angle θ(a,b)=77.6°. These dimensions conform with a commensurate (7 4|1 10) superstructure (***a*** **=**1.555 nm, ***b*** **=**2.438 nm, θ(a,b)=80.08°, (Figure 3 a). Each unit cell contains two distinct molecular features and 66 Cu atoms.

**Figure 3:**
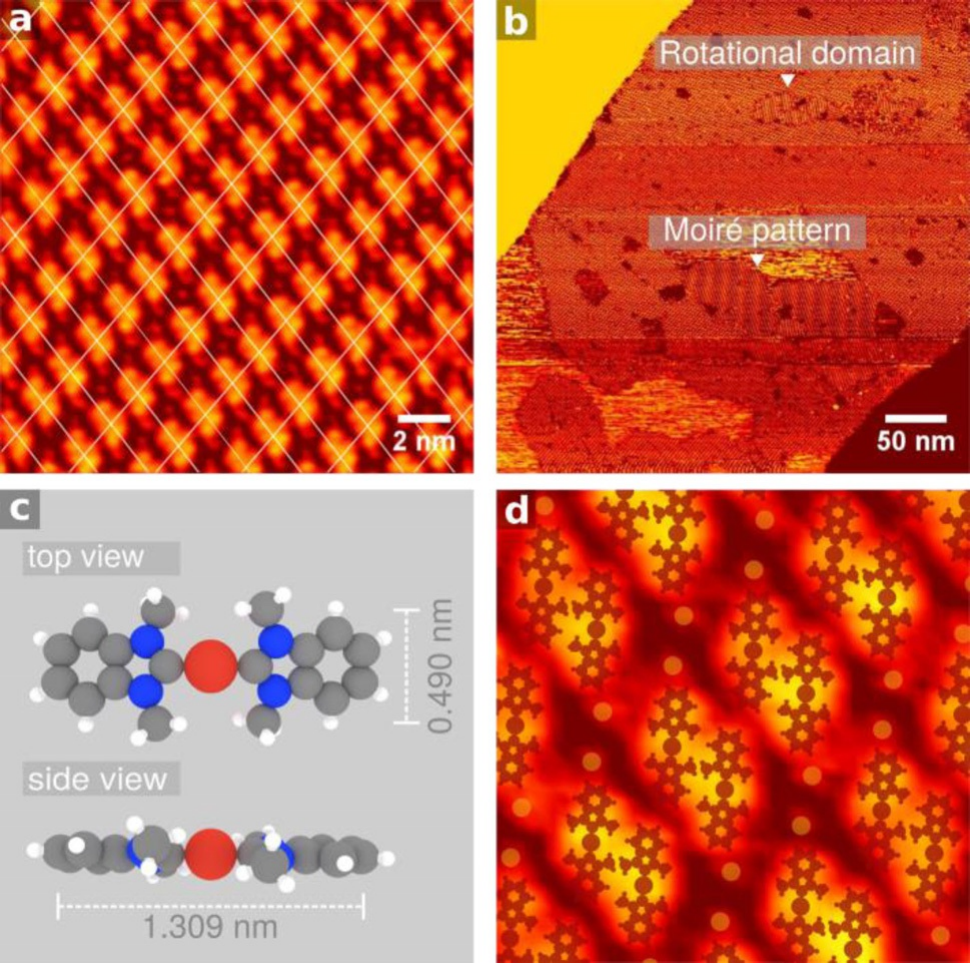
Thermally induced formation of (NHC)_2_Cu complex on Cu(111): a) High‐resolution image of features comprising the SAM formed upon annealing to 365 K; (7 4|1 10) unit cell is marked by overlaid grid. b) Large‐scale STM image of SAM. Domain boundaries and a Moiré pattern are visible. c) Molecular structure of (NHC)_2_Cu (gas phase). d) Proposed model of SAM in a) comprised of two (NHC)_2_Cu complexes (overlaid) intercalated by Cu adatoms (orange dots).

The SAM is dynamic in nature, although less so than its precursor prior to annealing. Reflectional and rotational domains, and a Moiré pattern were identified. Additionally, in situ defect correction and growth of a predominant domain were visible at 300 K (Figure 3 b and Figure S2). The growth of a preferential domain is likely directed by the crystallographic direction of steps at which the SAM nucleates. The features observed are too large to be attributed to single NHC molecules or enetetramines, and are instead assigned to pairs of (NHC)_2_Cu complexes coadsorbed with Cu adatoms (Figure 3 c, d).

Extraction of Cu atoms from steps and incorporation into molecular assemblies is a thermally activated process.^19^ Lifting of one atom from the (111) surface plane has precedent in the Au chemistry of isonitriles^22^ and thiols^3^, with recent studies reporting similar effects for NHCs.^7^ Rodríguez‐Castillo et al. concluded from DFT calculations that *upright* NHCs restructure Au surface atoms, probably as an intermediate step in the formation of (NHC)_2_Au^I^ complexes.^23^ Additionally, Tang and Jiang recently reported that (NHC)_2_Au complexes containing less sterically bulky NHCs favor flat lying NHC geometries driven by vdW interactions between the NHCs and the Au surface.^24^ Furthermore, (NHC)_2_Cu^0^ complexes have been isolated and characterized.^25^ Further work (e.g. XPS) would be required to identify the formal oxidation state of Cu in these complexes. Some evidence was found for ordered arrangements of NHC **1** on Au(111) (Figure S4), though the species were too mobile to image clearly. No ordering of NHC **3** was found on either Cu(111) or Au(111) at any coverage when imaging at 300 K. It is likely that the upright NHCs exhibit similar “ballbot‐type motion” to that described by Glorius and Fuchs for NHCs on Au.^7^ Essentially, upright NHC 3 skates around the surface riding on top of a Cu adatom. Glorius and Fuchs reported high mobility when imaging even at 77 K.^7^

TPD data further confirmed that the two types of adsorbed NHCs display distinctly different behavior. While *upright* NHC **3** desorbed cleanly from both Cu(111) and Au(111), *flat‐lying* NHCs **1** and **2** underwent more complex decomposition and desorption processes (See Figure S4 for data on Au). Figure 4 a shows coverage dependent TPD spectra for *m*/*z=*39 (C_3_H_3_^+^ from the benzene moiety) following the adsorption of NHC **3** onto Cu(111) at 300 K.

**Figure 4:**
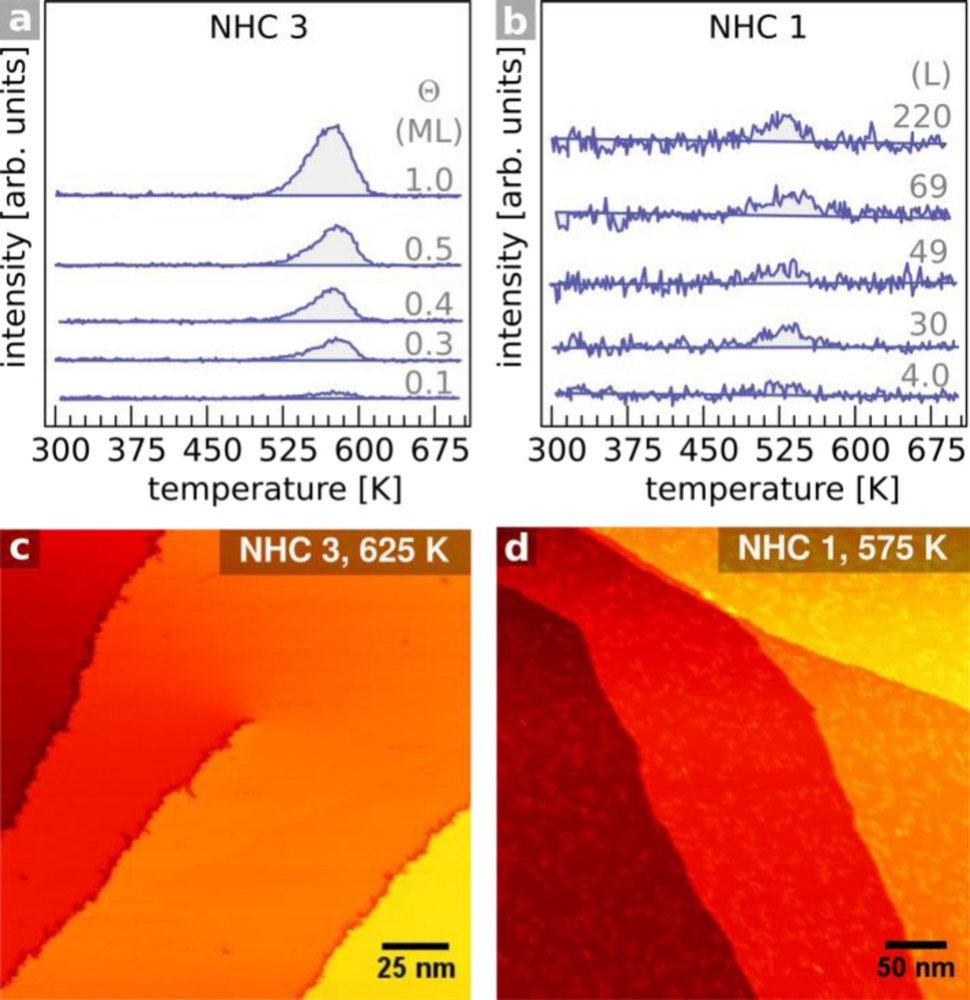
Thermally treated NHC films on Cu(111). TPD traces of fragment *m*/*z=*39 (C_3_H_3_^+^) of: a) NHC **3** and b) NHC **1**, *β*=2.1 Ks^−1^. STM images after high‐temperature heating of films prepared from: c) NHC **3** and d) NHC **1**.

Desorption occurred in a single peak (T_max_=570 K) independent of coverage; typical of first‐order kinetics.^26^ Coincident desorptions of H_2_, HCN, and C_3_H_5_^+^ (assigned to isopropyl groups) were also detected (Figure S3), suggesting that NHC **3** desorbs intact from Cu(111). A Redhead analysis yields a desorption energy of 152±10 kJmol^−1^; indistinguishable within error from the reported value of 158±10 kJmol^−1^ on Au(111).^9^, ^27^ DFT analysis of a single NHC **3** species on a Cu(111) slab predicts a binding energy higher than that observed on Au (182.6 kJmol^−1^ (see SI)). However, errors in such calculations are typically ≈20 kJmol^−1^, so theory and experiment are mutually consistent within error. Repulsive lateral interactions (not accounted for by the DFT calculations) may explain the lower value measured by TPD, though the fact that T_max_ is independent of coverage points to such interactions being relatively weak. These results confirm that NHCs form highly stable SAMs on Cu surfaces.

For films derived from NHC **1**, H_2_ evolution (*m*/*z=*2) occurs in two overlapping peaks at 532 K and 590 K which can be assigned to sequential thermally activated dehydrogenation steps.^28^ Desorption of HCN (*m*/*z=*27) occurs concurrently with H_2_ evolution, signifying that these desorption events are related. Unlike the TPD spectra for NHC **3**, the relative intensity of the fragments did not correlate with the exposure, suggesting a more complex surface chemistry. The yield for *m*/*z=*39 is marginal, implying that the ring moiety remains adsorbed (Figure 4 b). It can be concluded that NHC **1** is stable on Cu(111) up to the onset temperature for the first dehydrogenation step (≈475 K), contrasting starkly with the behavior of NHC **3**.

STM imaging of Cu(111) terraces after heating NHC **3** films resulted in clean surfaces, consistent with NHC desorption via a simple C‐Cu bond cleavage (Figure 4 c). In contrast, annealing NHC **1** and **2** films at high temperatures showed evidence for decomposition products, indicating a complex decomposition of *flat‐lying* NHCs (Figure 4 d). TPD and STM imaging of films derived from **2** (Figure S3) resembled closely the behavior observed for **1**, and an analogous interpretation is proposed. Thermal stability of the SAMs was also assessed by HREELS (Figure S1). Overall attenuation of all spectral features occurs above 560 K for all NHCs, consistent with the TPD findings.

In conclusion, the substituents at the *N*,*N*‐positions of the NHC are critical in determining the adsorption geometry and fate of the NHC‐based SAMs on Au and Cu. Small NHCs with dimethyl substituents form films with mixtures of *flat* and *upright* orientations, while the diisopropyl NHC stands *upright* only. The adsorption energy of NHC **3** on Cu(111) was found to be the same as on Au(111) within error. *Upright* NHCs desorb cleanly, while *flat‐lying* NHC films dissociate leaving surface contamination. For NHC **1**, ordered arrangements of (NHC)_2_Cu complexes were imaged. The fact that appropriately designed NHCs bind to Cu with high bond energies is a significant discovery and paves the way for future work on the practical applications of this surface functionalization. This work is currently ongoing in our laboratories.

## Experimental Section

See the Supporting Information for experimental details.

## Conflict of interest

*The authors declare no conflict of interest*.

## Supporting information

As a service to our authors and readers, this journal provides supporting information supplied by the authors. Such materials are peer reviewed and may be re‐organized for online delivery, but are not copy‐edited or typeset. Technical support issues arising from supporting information (other than missing files) should be addressed to the authors.

Supplementary
