# Supplementary material for: N‐Heterocyclic Carbene Self‐assembled Monolayers on Copper and Gold: Dramatic Effect of Wingtip Groups on Binding, Orientation and Assembly
Source: Chemphyschem. 2017 Nov 2;18(24):3536–9. doi: 10.1002/cphc.201701045 (PMC5765499; doi:10.1002/cphc.201701045)
Supplement: Supplementary file 1 — Supplementary [file CPHC-18-3536-s001.pdf]

CHEMPHYSCHEM

Supporting Information

N-Heterocyclic Carbene Self-assembled Monolayers on Copper and Gold: Dramatic Effect of Wingtip Groups on Binding, Orientation and Assembly

Christian R. Larrea,[a] Christopher J. Baddeley,*[a] Mina R. Narouz,[b] Nicholas J. Mosey,[b] J. Hugh Horton,[b] and Cathleen M. Crudden[b, c]

cphc_201701045_sm_miscellaneous_information.pdf

Contents

1 Preparation of NHC 1 1

2 Preparation of NHC 2 2

3 Vapour-deposition of NHCs 2

4 Computational details 3

5 Supplementary Figures: NHCs on Cu(111) 4

6 Supplementary Figures: NHCs on Au(111) 8

1 Preparation of NHC 1

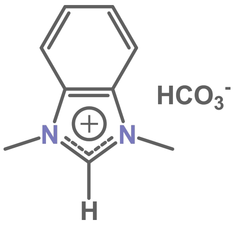

(1,3–Dimethyl–1H–benzo[d]imidazol–3–ium hydrogen carbonate)
1,3–Dimethyl–1H–benzo[d]imidazol–3–ium iodide was prepared according to literature procedures.[1] Hydrogen carbonate–anion exchange resin (Resin–HCO_3, 0.8 mmol HCO_3^-/1 mL resin) was prepared from Amberlyst A26 hydroxide resin as described in the literature.[2] Resin–HCO_3 suspended in water was measured out in a graduated cylinder (9 mL, 3 equiv) and transferred to a 50 mL flask where the resin was allowed to settle and water was decanted. The resin was washed with methanol (3 x 10 mL). 1,3–dimethyl–1H–benzo[d]imidazol–3-ium iodide (658 mg, 2.4 mmol) was dissolved in 12 mL methanol and transferred to the resin. The mixture was stirred for 30 min. The hydrogen carbonate solution was filtered to remove any resin beads and the resin was washed with methanol (3 x 5 mL), which was then added to the original filtrate. Solvent was evaporated and the residual solid was triturated and sonicated in acetone (3 x 10 mL), which was then decanted off *via* syringe and discarded. Subsequent drying of the white powder under vacuum afforded the desired product as a white powder (423 mg, 85 % yield). Anal. Calc. for $C_{10}H_{12}N_2O_3$: C, 57.68; H, 5.81; N, 13.45. Found: C, 58.21; H, 5.80; N, 13.31. ^{1}H NMR (300 MHz, CD_3OD): δ 7.95 (dd, J = 6.3, 3.1 Hz, 2H, Ar$-H$), 7.74 (dd, J = 6.3, 3.1 Hz, 2H, Ar$-H$), 4.14 (s, 3H, NCH_3). The N_2CH and HCO_3^- protons could not be observed due to their rapid exchange with the deuterated solvent on the NMR time scale. ^{13}C (^{1}H) NMR (125 MHz MHz, CD_3OD):

161.34 (s, HCO_3^-), 144.09 (s, $N_2\mathit{C}H$), 133.52 (s, C_q), 128.14 (s, C_{Ar}), 114.15 (s, C_{Ar}), 33.67 (s, $\mathit{C}H_3$).

2 Preparation of NHC 2

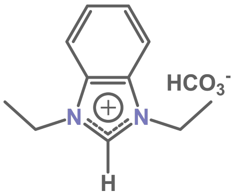

(1,3–Diethyl–1H–benzo[d]imidazol–3-ium hydrogen carbonate)

1,3–Diethyl–1H–benzo[d]imidazol–3–ium iodide was prepared according to literature procedures.[2] Resin–HCO_3 suspended in water was measured out in a graduated cylinder (12 mL, 3 equiv) and transferred to a 50 mL flask where the resin was allowed to settle and water was decanted. The resin was washed with methanol (3 x 10 mL). 1,3–diethyl–1H–benzo[d]imidazol–3–ium iodide (1 g, 3.31 mmol) was dissolved in 17 mL methanol and transferred to the resin. The mixture was stirred for 30 min. The hydrogen carbonate solution was filtered to remove any resin beads and the resin was washed with methanol (3 x 5 mL), which was then added to the original filtrate. Solvent was evaporated and the residual solid was triturated and sonicated in acetone (3 x 10 mL), which was then decanted off *via* syringe and discarded. Subsequent drying of the white powder under vacuum afforded the desired product as a white powder (662 mg, 85 % yield). Anal. Calc. for $C_{14}H_{20}N_2O_3$: C, 61.00; H, 6.83; N, 11.86. Found: C, 61.19; H, 6.83; N, 11.92. ^{1}H NMR (300 MHz, CD_3OD): δ 7.99 (dd, J = 6.3, 3.1 Hz, 2H, Ar$-\mathit{H}$), 7.71 (dd, J = 6.3, 3.1 Hz, 2H, Ar$-\mathit{H}$), 4.56 (q, J = 7.3 Hz, 4H, $NC\mathit{H}_2CH_3$), 1.65 (t, J = 7.3 Hz, 6 H, $-CH_2C\mathit{H}_3$). The $N_2C\mathit{H}$ and $\mathit{H}CO_3^-$ protons could not be observed due to their rapid exchange with the deuterated solvent on the NMR time scale. ^{13}C (^{1}H) NMR (75 MHz, CD_3OD): 161.37 (s, $H\mathit{C}O_3^-$), 142.07 (s, $N_2\mathit{C}H$), 132.84 (s, Cq), 128.43 (s, CAr), 114.31 (s, C_{Ar}), 43.40 (s, $\mathit{C}H_2CH_3$), 14.68 (s, $CH_2\mathit{C}H_3$).

3 Vapour-deposition of NHCs

Experiments were conducted in three separate stainless steel UHV chambers hosting an Ar ion sputtering gun and annealing facilities for sample cleaning. TPD data were collected in a UHV chamber (base pressure P = 3×10^{-10} mbar) equipped with a quadrupole mass spectrometer (SPECTRA, Microvision Plus) in direct line-of-sight with the crystal, and a LEED/Auger spectrometer (SpectaLEED, Omicron). HREELS experiments were conducted in a second chamber (base pressure P = 5×10^{-11} mbar)

hosting a double pass HREEL Ibach HIB1000 spectrometer ($E_0 = 5$ eV) separated from an adjacent preparation chamber. STM images were recorded in a third chamber (base pressure $P = 1 \times 10^{-10}$ mbar) equipped with a variable temperature scanning tunneling microscope (Omicron), and LEED optics. An electrochemically etched W tip was used for STM imaging typically employing bias voltages of $V_t = \pm 0.6$ V and tunneling currents of $I_t = 300$ pA. Image processing (plane substraction, equalisation, and FFT-filter) have been applied to the STM data using WSxM.[3] Error bars in distances are reported as standard deviations. Adsorbate unit cells are reported relative to a primitive surface unit cell described by unit vectors oriented along close packed surface directions related by a 120° rotation. Length of features were measured at the full width half maximum of their line profile. A Cu(111) single crystal was cleaned by cycles of annealing at 800 K and Ar ion sputtering (1.55 kV). Each cleaning cycle was terminated by annealing to 800 K for 15 min before the crystal was cooled down to room temperature. Annealing was done by either direct or resistive heating of the sample, and the temperature monitored by means of a type-K thermocouple. Cleanliness of the sample was assessed either by monitoring the TPD traces during the final annealing cycle until no desorbing species were observed, by HREELS until a featureless spectrum was obtained, or by STM and LEED until a sharp hexagonal (1x1) pattern and a virtually featureless topography was visible. Vapour deposition of NHC precursors was carried out by resistively heating a glass microcapillary hosting the chemical. Temperatures employed in the sublimation of **1**, **2**, and **3** were $T_{sub} = 335$, 325 and 325 K, respectively. We define 1 ML and submonolayer thereof as the integrated area of the saturated peak at $T_{max} = 570$ K of the TPD spectra.

4 Computational details

Density functional theory (DFT) calculations were performed to examine the structural features and binding energies of **3** on the copper slabs representing the (111) surface. To construct the slabs, the fcc unit cell of copper was optimized according to the method described below. A (111) surface was cleaved from the bulk structure and the resulting hexagonal cell was repeated twice in the lateral directions. The slabs used in the calculations were four layers thick, which tests showed was sufficient to converge surface energies to better than 1 mJ/m^2. The monomer was then added to the upper surface of the slab at positions corresponding to a-top site. The structure was relaxed while keeping the positions of the copper atoms in the bottom two layers of the slab fixed at their bulk positions. Analogous calculations were performed on the bare slab, i.e. the atoms in the upper two layers were relaxed while keeping those in the bottom two layers at fixed positions, and on the monomer, where all atoms were allowed to relax. The heights of the cells used in the calculations with the slab models were selected to ensure that at least 10 Å of vacuum space was present between periodic images. In addition, dipole correction techniques[4] were employed to eliminate spurious electrostatic interactions between periodic images along the direction normal to the slabs. All DFT calculations were performed using the PBEsol exchange correlation functional.[5] Core electrons were treated with projector augmented wavefunction potentials[6] including scalar relativistic effects on all atoms. The valence states were

represented with a planewave basis set expanded up to a kinetic energy cutoff of 40 Ry, and a kinetic energy cutoff of 400 Ry was used to represent the augmentation charges. A $3 \times 3 \times 1$ set of k–points was used in the calculations involving slabs. These details were sufficient to converge the total energies of the systems examined to better than 1 mV atom^{-1}. All calculations were performed with the Quantum-Espresso simulation package.[7]

The assignment of the vibrational modes is based on the computed spectra of model NHC – Cu – Cl complexes based on NHC **1-3** in the gas phase. The geometry of the modelled complexes were optimized and IR spectra computed using the M062X[8] functional implemented in the Gaussian09 package[9] at the default convergence criteria. The LANL2DZ[10] basis set was employed on all atoms. The presented calculated spectrum is not corrected for anharmonicity. Molecular building and visualisation of the vibrational modes were done using Gaussview.

5 Supplementary Figures: NHCs on Cu(111)

HREELS

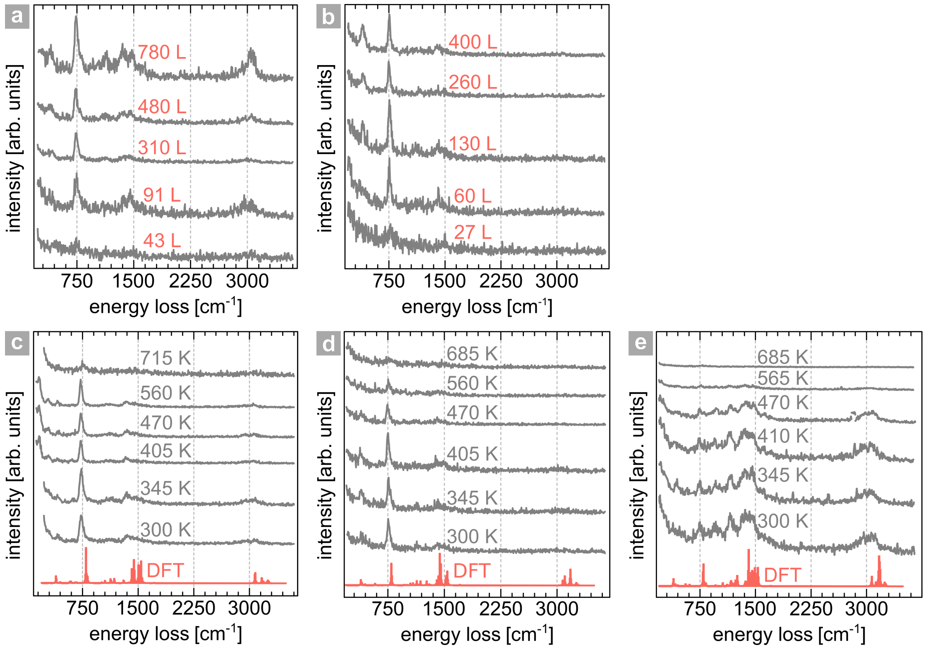

Figure S1: HREEL spectra of NHCs deposited onto Cu(111). a) NHC **1** and b) NHC **2** on Cu(111) as a function of increasing exposure in Langmuirs (1 L = 1×10^{-6} torr s) at 300 K. Spectra of NHC **1** show an increase in the intensity of the peaks in the 3000 cm^{-1} and 1000-1600 cm^{-1} region relative to the 750 cm^{-1} energy loss. This increase is associated with a higher concentration of *upright* species. Thermal stability of c) NHC **1** (120 L) d) NHC **2** (130 L) and e) NHC **3** (330 L) films probed by systematic annealing to higher temperature and HREEL spectra collected at 300 K, E_0 = 5 eV. The assignment of the energy losses in the spectra was carried out by comparison to the simulated normal vibrational modes of model NHC – M – Cl, (M = Cu, Au) complexes. Note that the calculated spectra contain contributions from M – Cl below 500 cm^{-1}.

Table S 1: HREEL spectra assignment of NHCs on Cu(111) based on NHC – Cu – Cl complexes of **1-3** modelled in the gas phase. Legend: β: bending, ω: wagging, τ: twisting, ν: stretching, δ: scissoring, *sym*: symmetric, *asym*: asymmetric, *ip*: in plane, *op*: out of plane.

NHC 1	NHC 2	NHC 3	assignment
3075	3000	3095	ν(C – H)$_{ring}$
2940		2980	ν(C – H)$_{alkyl}$
		1620	ring deformation
1460	1500	1480	ν(C=C), ν(C – N), $\delta(CH_2)$, β(C – H), $\beta(CH_3)_{asym.}$
1350	1410	1365	ν(C=C), ν(C – N), $\tau(CH_2)$, $\omega(CH_2)$, $\beta(CH_3)_{sym.}$
1130	1160	1165	ring β(C – H)$_{i.p}$
1030		960	ring breathing
730	735	755	ring β(C – H)$_{o.p}$
610		580	ring stretch
410	395	430	ring deformation/Cu – NHC
290			surface – $(NHC)_2Cu$
165			surface – $(NHC)_2Cu$

STM

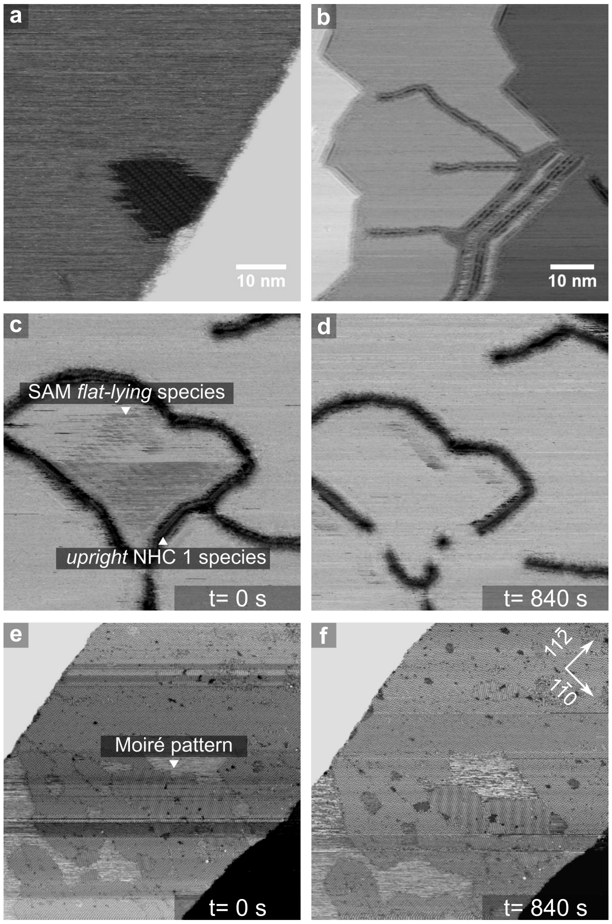

Figure S2: STM images of NHC **1** deposited onto Cu(111) a) small island of dimeric **1** on Cu(111) at 300 K, V_t = –0.6 V, I_t = 300 pA. b) trenches etched into the surface and *upright* NHC **1** molecules adsorbed at the edges, V_t = 0.6 V, I_t = 300 pA. These trenches branch off nodal points such as surface steps, screw dislocations, and etch pits and other trenches. c–d) trenches enclosing SAM of *flat–lying* species and NHC **1** hosted in trenches (300 K), V_t = –0.3 V, I_t = 600 pA. Consecutive topographs show healing of vacancies (Image size 41.3 × 41.3 nm^2). e–f) rotational, translational, and reflectional domains progression over time of $(NHC)_2Cu$ complexes and preferential growth of a domain aligned along the $<11\bar{2}>$ termination of a step. Also shown is a Moiré pattern of periodicity 13.5 nm, V_t = –0.6 V, I_t = 300 pA (image size 380 × 380 nm^2).

TPD

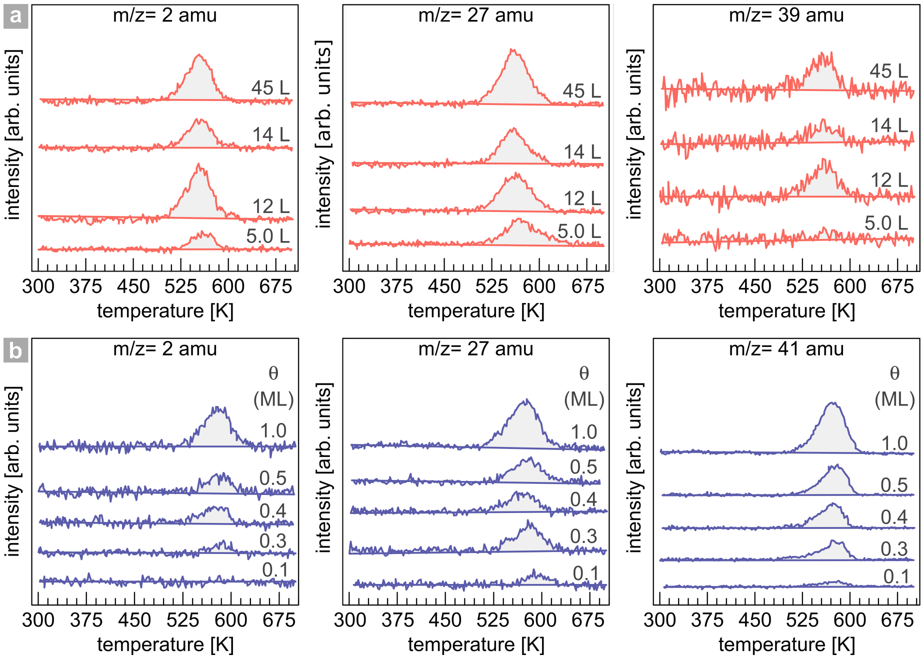

Figure S3: TPD spectra of NHCs deposited onto Cu(111) Fragments detected from the desorption of a) NH C **2**, $H_2^{+\bullet}$ (m/z = 2), HCN^+ (m/z = 27), and $C_3H_3^+$ (m/z = 39) and b) NHC **3**, $H_2^{+\bullet}$ (m/z = 2), HCN^+ (m/z = 27), and $C_3H_5^+$ (m/z = 41), $\beta = 2.1\ Ks^{-1}$.

6 Supplementary Figures: NHCs on Au(111)

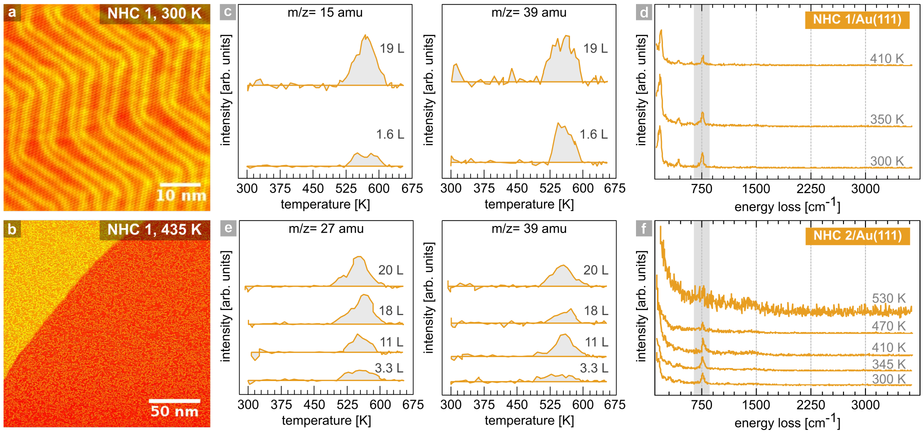

Figure S4: NHCs on Au(111). a) FFT–filtered STM image of NHC **1** on Au(111) at 300 K and b) decomposition products imaged after heating the sample to 435 K c) TPD spectra showed marginal desorption of m/z = 15 (CH_3^+) and 39 ($C_3H_3^+$) after deposition of NHC **1** d) HREEL spectra of NHC **1** (600 L) as a function of temperature analysed at 300 K. e) TPD spectra of NHC **2** shows desorption of fragments m/z = 27 (HCN^+) and 39 ($C_3H_3^+$) f) HREEL spectra of NHC **2** (530 L) as a function of annealing temperature and analysed at 300 K. Assignment of modes as in Table S1 for NHCs on Cu(111).

References

[1] W.-C. Chen, Y.-C. Lai, W.-C. Shih, M.-S. Yu, G. Yap, T.-G. Ong, *Chem. Eur. J.* **2014**, *20*, 8099–8105.

[2] C. M. Crudden, J. H. Horton, M. R. Narouz, Z. Li, C. A. Smith, K. Munro, C. J. Baddeley, C. R. Larrea, B. Drevniok, B. Thanabalasingam, A. B. McLean, O. V. Zenkina, I. I. Ebralidze, Z. She, H.-B. Kraatz, N. J. Mosey, L. N. Saunders, A. Yagi, *Nat. Commun.* **2016**, *7*, 12654.

[3] I. Horcas, R. Fernández, J. Gomez-Rodriguez, J. Colchero, J. Gómez-Herrero, A. Baro, *Rev. Sci. Instrum.* **2007**, *78*, 013705.

[4] L. Bengtsson, *Phys. Rev. B: Condens. Matter* **1999**, *59*, 12301.

[5] J. P. Perdew, A. Ruzsinszky, G. I. Csonka, O. A. Vydrov, G. E. Scuseria, L. A. Constantin, X. Zhou, K. Burke, *Phys. Rev. Lett.* **2008**, *100*, 136406.

[6] P. E. Blöchl, *Phys. Rev. B: Condens. Matter* **1994**, *50*, 17953.

[7] P. Giannozzi, S. Baroni, N. Bonini, M. Calandra, R. Car, C. Cavazzoni, D. Ceresoli, G. L. Chiarotti, M. Cococcioni, I. Dabo, *J. Phys.: Condens. Matter* **2009**, *21*, 395502.

[8] Y. Zhao, D. G. Truhlar, *Theor. Chem. Acc.* **2008**, *120*, 215–241.

[9] R. A. Gaussian09, *Inc. Wallingford CT* **2009**.

[10] P. J. Hay, W. R. Wadt, *J. Chem. Phys.* **1985**, *82*, 299–310.
